# Another Warning to Landlords

**Published:** 1890-06

**Authors:** 


### Another Warning to Landlords.

The law in respect to the liability of landlords for illness caused by defective sanitary arrangements is gradually becoming defined, and this will doubtless lead to more care being shown in the future in such matters, for the pocket is often more sensitive to responsibilities of this kind than the conscience. In an action tried a few days since at Tiverton a workman claimed damages from his landlady on account of an outbreak of typhoid fever in his house, which was ascertained to be due to the pollution of the water supply in consequence of defective drainage. The condition, indeed, was about as bad as it could be, one of the drains being blocked and the sewer discharging into the well, and the neglect was aggravated by the fact that on being complained to, the representatives of the landlady only made some minor and utterly inadequate repairs. A verdict was found for the plaintiff with £50 damages, a by no means exhorbitant compensation for the death of one child and the seriousness illness of the plaintiff and several other children.—*Medical Press and Circular.*

GLYCERINE MOUNTS THAT WILL KEEP.—Glycerine is a very desirable mounting medium for many purposes, and has but one drawback, and that is its tendency to creep out of the cells. When mounting such substances as will admit of such a procedure, I overcome this difficulty by using less glycerine than is required to fill the cells. This should be placed in the center of the cell, so that a circle of air will surround it in the finished mount. A coating of cement can be run on the cover glass over the circle of air, so that it does not show, but gives the mount the appearance of one with the cell full of glycerine.—*Formulary and Drug.*

COME HIGH BUT WE MUST HAVE THEM.—" What are your charges, doctor?"

" Three dollars a visit."

" Well, we don't want you to come on a visit, but just to stay ten or fifteen minutes."—*Puck.*

Effects of Close Shaving.—A writer in the *Med. Classics* looked through a microscope at a closely shaved face and he reports that the skin resembled a piece of raw beef. "To make the skin perfectly smooth requires," he says, "not only the removal of the hair, but also a portion of the cuticle, and a close shave means the removal of a layer of skin all around. The bloodvessels thus exposed are not visbile to the eye, but under the microscope each little quivering mouth holding a minute blood drop protests against such treatment. The nerve tips are also uncovered and the pores are left unprotected, which makes the skin tender and unhealthy. This sudden exposure of the inner layer of the skin renders a person liable to have colds, hoarseness and sore throat.

How to Clean Hypodermic Syringes.—The *Deutsch. Med. Wochenschr.*, 1889, says: Syringes whose canals have become obstructed so that a fine wire can not be drawn through them, are cleansed by holding them a moment over a flame; the foreign substance is thus quickly destroyed and driven off. If a wire has been rusted into the needle it should be dipped in oil before holding over the flame. To remove the rust from the interior of the cannla it is well to pass oil through the canula, then heating it; then rinse it out with alcohol.—*Pacific Record.*

Local committees of women in all the large Eastern cities are actively engaged in endeavoring to secure contributions of money for the purpose of founding a medical school in connection with the John Hopkins University of Baltimore, Md., at which women may study medicine as well as the men. It is expected that all the committees will turn in at least $100,000 by June 1.

A Dental Outpatients' Department in connection with the University of Vienna was opened April 21. The new institution is under the direction of Dr. Julius Scheff, and is the first step towards the establishment of a fully equipped school of dentistry in the Austrian capital.

